# Imagine Being Humble: Integrating Imagined Intergroup Contact and Cultural Humility to Foster Inclusive Intergroup Relations

**DOI:** 10.3390/bs14010051

**Published:** 2024-01-14

**Authors:** Emilio Paolo Visintin, Marika Rullo, Calogero Lo Destro

**Affiliations:** 1 Department of Humanities, University of Ferrara, 44121 Ferrara, Italy; 2 Department of Social, Political and Cognitive Science, University of Siena, 52100 Arezzo, Italy; marika.rullo@unisi.it; 3 Department of Psychology, Niccolò Cusano University, 00166 Rome, Italy; calogero.lodestro@unicusano.it

**Keywords:** imagined intergroup contact, cultural humility, intergroup anxiety, prejudice, future contact intentions, intergroup relations

## Abstract

To reduce prejudice and to promote intergroup harmony and equality, the imagined intergroup contact technique, based on the mental simulation of an encounter with an outgroup member, has been proposed. Though a substantial body of research has provided support for the efficacy of imagined intergroup contact in prejudice reduction, an alternative strand of research has raised questions about its effectiveness. In this experiment, we combined imagined intergroup contact with cultural humility, that is, an other-oriented, humble approach toward people with different cultural backgrounds, recognizing status and power imbalances and privileges. Specifically, we tested whether instructions aimed at eliciting cultural humility during imagined contact boosted its effectiveness in reducing prejudice and promoting future contact intentions, compared to a standard imagined contact condition and to a control imagination task. Intergroup anxiety was tested as a mediator of the effects of culturally humble imagined contact on reduced prejudice and on future contact intentions. We found that culturally humble imagined contact, compared to the two other conditions, reduced intergroup anxiety and yielded indirect effects on reduced prejudice and increased future contact intentions. The findings will be discussed by focusing on the integration of cultural humility in prejudice reduction techniques based on intergroup contact.

## 1. Introduction

Among the challenges of societies nowadays, reaching intergroup harmony and equality is one of the crucial issues. While ethnic and cultural diversity creates opportunities for intergroup contact, segregation, prejudice, and discrimination still persist. Therefore, social and behavioral scientists are investigating strategies to reduce prejudice and discrimination which do not require face-to-face encounters between members of different ethnic and cultural groups. Among the so-called indirect contact forms, i.e., forms of intergroup contact which do not require face-to-face interactions, imagined intergroup contact [1,2], i.e., the imagination of a (positive) encounter with a member of an outgroup, is considered an effective and easily implemented strategy to reduce prejudice and to foster intentions to have direct contact with outgroup members [3]. While there is ample empirical evidence supporting the effectiveness of imagined intergroup contact, some studies have failed to replicate these effects [4]. Additionally, other works have suggested the existence of boundary conditions of the imagined contact effect [5]. In this study, we propose that, to boost the effectiveness of imagined contact across people with different characteristics, imagined contact instructions could be integrated with the concept of cultural humility, which has been defined as “having an interpersonal stance that is other-oriented rather than self-focused, characterized by respect and lack of superiority toward an individual’s cultural background and experience” [6]. Recent research has shown that cultural humility is indeed associated with reduced prejudice toward several outgroups [7] and that cultural humility can also shape the impact of predictors of prejudice such as inegalitarian ideologies [8] and ethnic and cultural diversity [9]. While both imagined intergroup contact and cultural humility are associated with reduced prejudice and can promote intergroup harmony and equality, no previous study has combined them. In this experimental study, we aimed at filling this gap. Therefore, we proposed and tested whether instructions aimed at eliciting cultural humility during an imagined intergroup encounter can boost the effectiveness of imagined contact in prejudice reduction.

### 1.1. Imagined Intergroup Contact

Since the initial formulation of the contact hypothesis [10], a wealth of research has tested it and found that having contact with members of external groups (outgroups) is indeed associated with a reduction in different forms of prejudice [11,12]. However, direct intergroup contact is not always feasible for several reasons. First, people might not have the opportunity to interact with members of different groups because there might not be outgroup members in the area they live or because of segregation, as people tend to stick together with members of their ingroup despite having opportunities for intergroup contact [13,14]. Second, direct interactions with members of external groups, and especially the very first intergroup interactions, are likely to be anxiety-provoking [15,16], and intergroup anxiety constitutes a barrier to future contact experiences [17] and to prejudice reduction [18]. Also, while most direct contact situations are usually experienced as positive, some intergroup encounters might be experienced negatively, and negative contact is likely to increase prejudice [19]. 

Social and behavioral scientists have therefore considered the implementation of intergroup contact strategies that do not require face-to-face, direct contact. Indeed, indirect contact is likely to be less anxiety-provoking, given that people in indirect contact situations do not expect a face-to-face interaction with outgroup members [20]. Also, indirect contact strategies can be designed to ensure the positiveness and pleasantness of the contact experience. Since the initial formulation of the extended contact hypothesis [20], which posits that knowing an ingroup member who has an outgroup friend might reduce prejudice similarly to direct contact, different indirect contact strategies have been proposed. For example, research has distinguished between extended contact (previously defined) and vicarious contact, i.e., the observation of an intergroup encounter [21]. Both extended and vicarious contact, if positive, are likely to reduce prejudice. However, in extended and vicarious contact situations, the self is not involved in the intergroup encounter. 

Instead, in the imagined contact paradigm, which is used in the current study, the self is directly involved in the intergroup encounter, because imagined contact experiments and interventions are based on the imagination of the self while having a (positive) interaction with an outgroup member. A wealth of research has found that imagined intergroup contact can reduce prejudice [1], increase intentions of future contact with outgroup members [22], and promote cooperative behavior [23]. The effectiveness of imagined intergroup contact has also been demonstrated in a meta-analysis [3]. Noteworthily, imagined intergroup contact does not reduce prejudice only in the laboratory or in online experiments, but programs based on imagined contact have also been implemented in school settings. For example, Vezzali et al. [24] conducted a three-session imagined contact intervention in primary schools in Italy and found that the intervention was effective at reducing implicit prejudice and increasing positive behavioral intentions toward immigrants. Furthermore, Vezzali et al. [25] found that a multifaceted imagined contact intervention in Italian primary schools could be used to counteract discriminatory bullying, because the intervention increased intentions to counteract bullying and social exclusion of children with disabilities.

Despite the widespread support for the imagined intergroup contact paradigm, the ease of implementation of the technique, and its potential to be incorporated in field interventions, some scholars have expressed skepticism about imagined contact and pointed out the limitations of the paradigm [26]. Indeed, there are published reports of failures of imagined contact to reduce prejudice, e.g., [4]. Other research has shown that the effects of imagined intergroup contact might not be universal, suggesting that they may instead be limited to certain individuals or be moderated by characteristics of the imagined contact situation. For example, it has been found that imagined contact is especially effective at improving outgroup attitudes among prejudiced individuals [27,28]. Among the characteristics of the imagined contact task, there is evidence that imagined contact effects might be boosted, for example, by instructions aimed at increasing elaboration [22], by counter-stereotypical characteristics of the outgroup member [29], and by imagining the physical touching of hands [30].

Noteworthily for the current research, the mutual intergroup differentiation model [31,32] proposes that intergroup contact is most effective at reducing prejudice when there is a salience of intergroup boundaries, meaning that it is clear that contact occurs between members of different groups, and/or when the outgroup member in the contact situation is typical of their group. Indeed, intergroup salience and/or outgroup typicality are necessary to generalize the positive attitude from the encountered outgroup member to the whole outgroup. Otherwise, if intergroup contact occurs at the purely individual level and there is no awareness of group belongings, it is unlikely that positive attitudes will generalize to the whole outgroup [33]. Research supports the mutual intergroup differentiation model, indicating that contact is more effective at reducing prejudice when intergroup boundaries are salient and when the outgroup member involved in the contact situation is representative of their group [34]. In line with these notions, research employing the imagined contact paradigm has also found beneficial effects of intergroup salience and of outgroup typicality. For example, Pagotto et al. [23] found that focusing on intergroup differences during imagined contact improved outgroup attitudes and increased intergroup cooperation compared to a control condition and compared to imagined contact purely at the individual level. Similarly, Stathi et al. [35] found that imagined contact is more effective when group belongings are salient and when the typicality of the outgroup member is high; see also [36,37].

In this study, we propose that instructions aimed at eliciting cultural humility during imagined contact could boost the effectiveness of imagined contact in reducing prejudice and increasing future contact intentions.

### 1.2. Cultural Humility

The concept of cultural humility has been proposed in healthcare settings with the idea that being culturally humble could help physicians, nurses, and psychotherapists to interact with people from different cultures [6]. Cultural humility is a subdomain of humility and refers to humility in the context of intercultural and intergroup relations. Like general humility, cultural humility also comprises two dimensions: an intrapersonal one, i.e., an accurate view of the self and the recognition of one’s strengths and weaknesses, and an interpersonal one, i.e., adopting a stance which is oriented toward the other rather than toward the self [7]. Cultural humility involves believing that one’s own culture is not superior to other cultures and recognizing that there are differences between cultures, without asserting the superiority of any particular culture. Cultural humility also implies the acknowledgement of status and power differences between cultural groups and the willingness to address such differences [38]. As there are several definitions and conceptualizations of cultural humility in the literature, Foronda et al. [39] conducted a conceptual analysis of the published literature on cultural humility and identified five key attributes of cultural humility: openness (i.e., being open to new ideas and to engaging in cross-cultural exchanges), self-awareness (i.e., awareness of one’s own strengths and weaknesses, biases, values), egoless (i.e., humbleness and modesty and belief that all human beings are equal), supportive interaction (i.e., willingness to engage in positive and supportive interactions with other people), and self-reflection and critique (i.e., engaging in a continuous process of deep introspection). 

While cultural humility training was originally proposed in healthcare settings to enhance the experiences of healthcare professionals and patients from diverse cultures [40,41], recent research has also highlighted the significance of cultural humility for the general population. For example, analyzing an American general population sample, Captari et al. [42] found that cultural humility, treated as an individual difference variable, was associated with openness toward immigration and less prejudice toward Syrian refugees. Cultural humility has also been found to be associated with tolerance toward religious outgroups [43] and toward sexual minorities [44] (for a review, see [7]). Visintin and Rullo [8] found that cultural humility was associated with reduced prejudice toward immigrants in Italy and that cultural humility also buffered the association between social dominance orientation, i.e., support for intergroup hierarchies [45], and prejudice against immigrants. Rullo et al. [9] replicated a negative association between cultural humility and prejudice against immigrants and against Muslims in Italy and further found that cultural humility favored the beneficial effects of perceived ethnic and cultural diversity, which was associated with more negative intergroup contact only among individuals with low cultural humility.

Noteworthily, to the best of our knowledge, no previous research has experimentally manipulated cultural humility. However, previous research suggests that humility can be primed or elicited in experimental settings. For example, Van Tongeren et al. [43] primed participants with humility-related words at the subliminal level, finding that such priming reduced aggressive behavior (i.e., supposedly administering hot sauce to another participant). 

The reviewed literature suggests that cultural humility is associated with reduced prejudice and that it might counteract the effects of antecedents of prejudice (e.g., intolerant ideologies). In this study, we further propose that cultural humility could be integrated into imagined contact to increase its effectiveness.

### 1.3. The Current Study

Previous failures to replicate the imagined contact effect, e.g., [4], and previous research identifying the boundary conditions of the effectiveness of imagined contact, e.g., [27,28], suggest that some modifications to instructions for imagined contact might enhance its effectiveness. In this research, we integrated the imagined contact paradigm and cultural humility and tested whether instructions aimed at eliciting cultural humility during the imagined interaction might strengthen the effectiveness of imagined contact in prejudice reduction. Our prediction is in line with the mutual intergroup differentiation model [31,32]. Indeed, being culturally humble means being open to discovering other cultures and to acknowledging and addressing power and status inequalities. To be open to discovering new cultures with a non-judgmental approach, and to recognize and confront power and status asymmetries, one needs to be aware of belonging to different cultural groups. Therefore, if an imagined encounter happens under instructions aimed at eliciting cultural humility, group belongings should be salient.

In an experiment we tested whether instructions aimed at eliciting cultural humility and at having a culturally humble approach during an imagined encounter could boost the effectiveness of imagined intergroup contact in reducing prejudice and fostering future contact intentions compared to standard imagined contact instructions and to control imagination tasks. 

We also tested the mediator role of intergroup anxiety in the effects of culturally humble imagined contact. Intergroup anxiety is defined as the anxiety associated with the idea of interacting with outgroup members [16,18]. Previous research on intergroup contact has found intergroup anxiety is the most reliable mediator of the contact-reduced prejudice association [46], meaning that intergroup contact reduces prejudice by reducing intergroup anxiety. The mediating mechanism of intergroup anxiety has also been well established in imagined contact research. For example, Turner et al. [1] found that, among heterosexual men, imagining an encounter with a gay man reduced intergroup anxiety and consequently prejudice (see also [47]). As cultural humility implies supportive, non-judgmental interactions with people from other cultures, it is expected to further reduce intergroup anxiety compared to standard imagined contact and control imagination tasks. Therefore, we expected the effects of culturally humble imagined contact on prejudice and on future contact intentions to be mediated by intergroup anxiety.

## 2. Materials and Methods

### 2.1. Participants

This research study employed a convenience sample of students from two universities in Italy. They participated in the online experiment to obtain partial course credit. Given that immigrants were the target outgroup, in data analysis, we only focused on participants with Italian nationality. Furthermore, we excluded participants who failed one or both attention checks (see procedure). The final sample included 464 Italian university students who completed the experiment. The mean age was 30.19 (standard deviation = 10.81). This mean age is likely higher compared to that of typical university students because one of the two Italian universities where data were collected is an online university attended by several working students older than typical university students. Regarding gender, 230 were females, 228 were males, 5 were non-binary, and 1 participant answered “other”.

### 2.2. Procedure

Potential participants were recruited through invitations to participate in a social psychology study via online teaching platforms. The experiment was conducted via Qualtrics. When students clicked on the link, they were first provided with all the information about the study and invited to provide informed consent to participate in the study. Next, they were randomly assigned to one out of three experimental conditions. In the standard imagined contact condition, the instructions were “Please take a few minutes to imagine meeting Mouna, an immigrant from Morocco on a train or on bus. The two of you spend about half an hour talking, until you get to your destination”. In the culturally humble imagined contact condition, the standard imagined contact condition instructions were integrated with the following sentence: “During the conversation, your approach is curious and open to discovering Mouna’s culture and cultural differences, aware that you still have a lot to learn about other cultures. Furthermore, the encounter allows you to reflect on privileges and inequalities between cultural groups in society”. In the control condition, instructions were the same as in the standard imagined contact condition, with the exception that the imagined encounter happened with Roberta, a female stranger (Roberta is a typical Italian female name). Participants could advance to the next question after two minutes. In the next question, they were invited to describe what they had imagined, to reinforce the experimental manipulation. Again, they could advance to the next set of questions after two minutes. Next, they answered a series of psychosocial measures.

Intergroup anxiety was measured with 6 items, with a response scale from 1 (not at all) to 7 (very much), adapted from Visintin et al. [48]. Participants were invited to think about meeting an immigrant stranger in the future, and to rate to what extent they would feel embarrassed, anxious, shy, competent (reverse-coded), relaxed (reverse-coded), and nervous. A preliminary inspection of data, when 140 answers from Italian participants were collected, suggested that one reverse-coded item (competent) had low factor loading and was replaced with another reverse-coded item (comfortable). After reverse-coding the appropriate answers, they were averaged to create a reliable (Cronbach’s alpha = 0.79) composite score, with higher values representing higher intergroup anxiety. Note that when running the data analysis with a 5-item composite score, i.e., excluding answers to the item which was replaced after the initial inspection of data and only considering items which were the same throughout the data collection, the results did not change. 

Prejudice was assessed with a single question, asking respondents to report their attitude toward immigrants on a response scale from 0 (extremely unfavorable) to 100 (extremely favorable) [8]. Answers were reverse-coded, so that higher values represent more prejudice.

Future contact intentions were assessed with five questions adapted from Husnu and Crisp [22], with a response scale from 1 (not at all) to 7 (very much). Sample questions are “Would you like to have more contact with immigrant people?” and “Would you be willing to spend some time to get to know immigrant people better?”. Answers were averaged to create a reliable (Cronbach’s alpha = 0.93) composite score, with higher values representing higher future contact intentions. 

The questionnaire included additional measures. Information about them can be obtained upon request from the corresponding author. 

In the preliminary inspection of data (where *n* = 140 Italian respondents), we also realized that some participants might not have paid enough attention to the task, as suggested by the long response time to the whole questionnaire or by short answers to the open question about the description of the imagined encounter. Therefore, we included two attention checks, one asking participants to pick the first option of the response scale and the other one asking participants to select the last option of the response scale. When analyzing the data of Italian participants who filled the questionnaire version including attention checks, 45 out of 369 failed one or both attention checks, and therefore they were excluded from data analysis. 

### 2.3. Data Analysis

We first calculated and reported descriptive statistics, i.e., means and standard deviations by experimental condition, and bi-variate correlations.

As two variables, i.e., intergroup anxiety and future contact intentions, were assessed by multiple items, we ran a confirmatory factor analysis (CFA) to test the empirical distinction between them. For both variables, we created three parcels combining subsets of items [49]. We tested a model with six observed variables and two latent variables. The CFA was run in Mplus. 

To test our predictions, we created two contrasts. The first one (contrast 1) contrasted the culturally humble imagined contact condition (coded +2) with the standard imagined contact and the control conditions (both coded −1). The first contrast allowed us to test our key novel hypothesis, i.e., that culturally humble imagined contact is more effective in reducing intergroup anxiety and prejudice and in fostering future contact intentions compared to a control condition and to standard imagined contact instructions. The second one (contrast 2) contrasted the standard imagined contact condition (coded +1) with the control condition (coded −1); the culturally humble imagined contact condition was coded 0. The second contrast had the goal of testing whether the standard imagined contact condition reduced intergroup anxiety and prejudice and increased future contact intentions compared to the control condition, i.e., it allowed us to test whether the standard imagined contact effect is replicated in our experiment. 

We first tested whether the two contrasts predicted intergroup anxiety, prejudice, and future contact intentions. Next, to test whether there were mediated/indirect effects of the two contrasts on prejudice and on future contact intentions via intergroup anxiety, we used the Process macro (Model 4) and bootstrapping with 10,000 resamples. In all regression and mediation/indirect effects analyses, the two contrasts were included simultaneously as predictors. 

To account for modifications in the procedure (replacement of an item in the intergroup anxiety measure and inclusion of attention checks), we created an additional contrast, where participants who answered the first version of the questionnaire (before replacing one intergroup anxiety item and without attention checks, *n* = 140) were coded −1 and participants who answered the second version of the questionnaire (after replacing the intergroup anxiety item and with attention checks, *n* = 324) were coded +1. Including this additional contrast in the regression analyses did not change the result pattern. Therefore, we present the results without such additional contrast.

In additional regression analyses, we also controlled for gender and age, and the results did not change. Finally, neither gender nor age interacted with contrast 1 or contrast 2. Therefore, the effects of the experimental manipulations did not vary as a function of gender or age. For the sake of simplicity, we report the results without such covariates and interactions. 

## 3. Results

### 3.1. Preliminary Results 

Means and standard deviations by experimental condition are reported in Table 1. Bi-variate correlations between measured variables are reported in Table 2.

In the CFA testing the empirical distinction between intergroup anxiety and future contact intentions, the model fitted the data well: χ^2^ (8) = 9.36, *p* = 0.312, root mean square error of approximation = 0.019, standardized root mean square residual = 0.025, comparative fit index = 0.99. Factor loadings were ≥0.581 and significant (*p*s < 0.001). The two latent variables were empirically distinct (*r* between latent variables = −0.20, standard error = 0.05). 

### 3.2. Main Results

As shown in Table 3, contrast 1 negatively predicted intergroup anxiety, indicating that intergroup anxiety was lower among participants in the culturally humble imagined contact condition compared to participants in the other two conditions. Instead, contrast 2 did not yield a significant effect on intergroup anxiety, suggesting that intergroup anxiety did not differ between participants in the standard imagined contact condition and those in the control condition.

Neither contrast 1 nor contrast 2 yielded significant effects on prejudice or on future contact intentions (*p*s > 0.41). Therefore, prejudice and future contact intentions did not differ as a function of the experimental manipulation.

While we could not test for mediation, as the predictors did not have significant effects on the dependent variables [50], we could test whether culturally humble imagined contact had indirect effects on prejudice and on future contact intentions via intergroup anxiety. In line with Hayes [51] and Aguinis et al. [52], it is possible to test whether culturally humble imagined contact reduced intergroup anxiety compared to standard imagined contact and to control conditions, and whether for those participants who had a reduction in intergroup anxiety following culturally humble imagined contact there is a significant probability that the lower their intergroup anxiety, the lower their prejudice, and the lower their intergroup anxiety, the higher their future contact intentions. 

**Figure 1:**
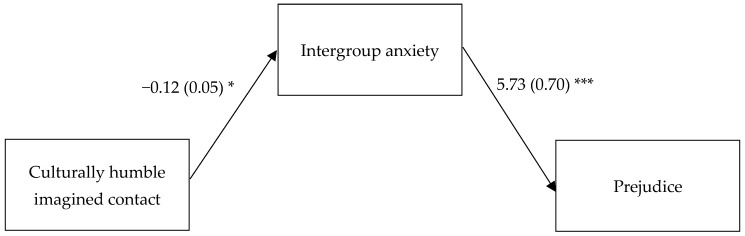
Indirect effect of culturally humble imagined contact on prejudice via intergroup anxiety. In the contrast representing the predictor, culturally humble imagined contact was coded +2 while standard imagined contact and control were coded −1. Contrast 2 (+1 = standard imagined contact, −1 = control; 0 = culturally humble imagined contact) was controlled for. The direct association between culturally humble imagined contact and prejudice was not significant (*B* = 0.06, *SE* = 0.84 *p* = 0.942). *** *p* < 0.001. * *p* < 0.05.

**Figure 2:**
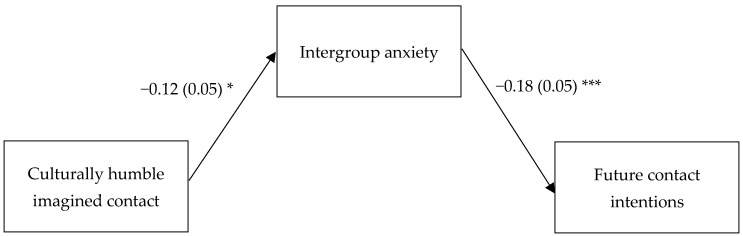
Indirect effect of culturally humble imagined contact on future contact intentions via intergroup anxiety. In the contrast representing the predictor, culturally humble imagined contact was coded +2 while standard imagined contact and control were coded −1. Contrast 2 (+1 = standard imagined contact, −1 = control; 0 = culturally humble imagined contact) was controlled for. The direct association between culturally humble imagined contact and future contact intentions was not significant (*B* = 0.03, *SE* = 0.06, *p* = 0.669). *** *p* < 0.001. * *p* < 0.05.

Consistently with our hypothesis, culturally humble imagined contact had a negative indirect effect via intergroup anxiety on prejudice (*B* = −0.68, *SE* (boot) = 0.32, 95% CI = [−1.355, −0.098]) and a positive indirect effect via intergroup anxiety on future contact intentions (*B* = 0.02, *SE* (boot) = 0.01, 95% CI = [0.004, 0.051]) (see Table 3 for regression coefficients and Figure 1 and Figure 2 for a graphical representation of the indirect effects).

## 4. Discussion

Prejudice reduction and future intergroup contact intentions are of paramount importance in fostering harmonious and inclusive societies. In this work, we tested whether instructions eliciting cultural humility boosted the effectiveness of imagined intergroup contact in fostering future intergroup contact intentions and in reducing prejudice compared to a standard imagined contact condition and to a control imagination task. We further investigated the mediating role of intergroup anxiety in the relationship between (culturally humble) imagined intergroup contact and both prejudice and future contact intentions as the dependent variables. In line with our predictions, culturally humble imagined contact reduced intergroup anxiety compared to standard imagined contact and to a control condition. Intergroup anxiety was in turn associated with higher prejudice and with lower future contact intentions; therefore, there were indirect effects of culturally humble imagined contact on prejudice and on future contact intentions via anxiety. However, there were no significant direct effects of culturally humble imagined contact on prejudice and on future contact intentions. Furthermore, there was no difference in any mediator or outcome variable between the standard imagined contact and the control condition. 

Our findings corroborate the importance of cultural humility for intergroup harmony and equality [7,8,42,44]. Indeed, cultural humility instructions contributed to the effectiveness of imagined contact in reducing intergroup anxiety and to indirect effects on prejudice and on future contact intentions via intergroup anxiety. Our findings further suggest that cultural humility can be easily elicited by simple instructions to be humble, non-judgmental, open to differences, and to consider and address status and power imbalances. This aligns with previous research which found that a subliminal priming of humility-related words could reduce aggressive behavior [43]. Therefore, we believe that, while complex and multi-session cultural humility training is valuable for healthcare professionals and the general population to increase tolerance and promote effective, supportive, and egalitarian intercultural communications, even simple inductions of cultural humility can contribute to intergroup harmony and equality. 

Our findings also shed light on how to structure imagined contact experiments and interventions. Indeed, we found that a brief and simple sentence aimed at eliciting cultural humility can contribute to the effectiveness of imagined contact in reducing intergroup anxiety and to its indirect effects on future contact intentions and reduced prejudice. Therefore, inducing cultural humility during imagined contact could be an easy-to-implement way to boost the effectiveness of imagined contact. However, caution should be taken about our findings. It is worth remarking that, despite the large sample, we did not find direct effects on the outcomes, i.e., prejudice and future contact intentions, but only indirect effects via intergroup anxiety. The main conclusion from this research is that cultural humility instructions during imagined contact were effective in reducing intergroup anxiety, which is per se a noteworthy result, given that intergroup anxiety represents a barrier to positive and egalitarian intergroup relations [17,18]. However, no firm conclusions can be drawn regarding prejudice reduction and behavioral intentions. Furthermore, we did not find support for the standard imagined contact effect because there was no difference on intergroup anxiety, prejudice, and future contact intentions between the standard imagined contact and the control condition. Future studies are needed to better understand the nature of these results. It is possible that, among university students who are likely to be relatively unprejudiced [53], standard imagined contact is not enough to change emotions, attitudes, and behavioral intentions. It is also possible that the implementation of imagined contact via online platforms is suboptimal, because there might be several distracting factors while people are asked to undertake the imagination tasks. 

### Limitations and Future Research Directions

Some limitations of this research need to be acknowledged. First, and as anticipated, we examined a convenience sample consisting solely of university students, who might represent a relatively unprejudiced sample [53]. This likely explains the very low levels of prejudice across the experimental conditions. Based on research showing that imagined contact is more effective among prejudiced people [28], it is possible that findings might be stronger when analyzing a general population sample which might have higher baseline prejudice. However, this needs to be tested in future research, and therefore we recommend future research to replicate our experiment with a representative sample of the general population. Furthermore, it would have been beneficial to assess intergroup anxiety, prejudice, and future contact intentions, and also previous intergroup contact experiences before the experimental or control tasks. This would have allowed us to control for such variables in the data analysis and to test whether there was an actual reduction in intergroup anxiety following culturally humble imagined contact. We also acknowledge that the mediator and the dependent variables were all measured at the same time point, just after the experimental and control tasks. While treating intergroup anxiety as the mediator of the effects of (imagined) contact is based on the literature [46], including also longitudinal studies, e.g., [54,55], it is not possible to establish causality between the mediators and the outcome variables. Additionally, with the current study’s design, we cannot determine the duration of the effects of culturally humble imagined contact on intergroup anxiety. Future research should employ longitudinal research designs. Furthermore, we only analyzed self-reported behavioral intentions, but did not assess actual behavior. While behavioral intentions are considered the most proximal predictor of behavior [56], future research integrating imagined contact and cultural humility should also measure actual behavior. Finally, we analyzed the point of view of only Italian respondents, imagining contact with a female immigrant from a specific nationality (Morocco). We decided to focus on an immigrant group which is present and stigmatized in the country of data collection. Future research should replicate our findings in other intergroup contexts to test their generalizability. We also decided to invite all participants to imagine an encounter with a female partner, to keep gender of the immigrant target consistent across the respondents and not to add further complexity to the research design, given that this is the first test of the combination of imagined contact and cultural humility. However, prejudice against immigrants might differ as a function of the gender of the immigrant target. On the one hand, as suggested by Ji et al. [57], males might possibly be the target of higher prejudice than females. On the other hand, an immigrant woman represents a gender ingroup member to female participants but a gender outgroup member to male participants. While controlling for the gender of the participants did not change our result pattern, and we did not find that the effects of imagined contact varied as a function of participant gender, we encourage future research to further investigate the role of gender of the imagined outgroup member as a function of the gender of each participant. Despite these limitations, our experiment suggests that integrating imagined contact and cultural humility might be useful in reducing intergroup anxiety, which represents the first step toward prejudice reduction and toward intergroup harmony and equality. 

The findings of the present study hold practical implications, suggesting that interventions designed to combine intergroup contact and cultural humility can play a crucial role in addressing social divisions and biases. Additionally, exploring the potential for tailored educational and training programs to instill cultural humility within various contexts, such as schools and workplaces, may offer valuable insights. To conclude, our research aims at opening the door for developing strategies based on intergroup contact and cultural humility to reduce intergroup anxiety and prejudice and to foster future contact intentions, with the ultimate goal of fostering inclusive, harmonious, and egalitarian intergroup relations. 

## Figures and Tables

**Table 1:** Means (and standard deviations) by experimental condition.

	Culturally Humble Imagined Contact (*n* = 139)	Standard Imagined Contact (*n* = 169)	Control (*n* = 156)
Intergroup anxiety	2.52 (1.03)	2.78 (1.10)	2.74 (1.14)
Prejudice	17.42 (18.80)	19.15 (17.67)	18.18 (16.69)
Future contact intentions	5.71 (1.28)	5.67 (1.19)	5.55 (1.26)

**Table 2:** Means, standard deviations (SD), and correlations between variables.

	Mean (SD)	1	2
1. Intergroup anxiety	2.69 (1.10)	-	
2. Prejudice	18.31 (17.68)	0.36 ***	-
3. Future contact intentions	5.64 (1.24)	−0.16 ***	−0.61 ***

Notes. *** *p* ≤ 0.001.

**Table 3:** Regression analyses predicting intergroup anxiety, prejudice, and future contact intentions.

	Intergroup Anxiety	Prejudice	Future Contact Intentions
Contrast 1	−0.12 (0.05) *	0.06 (0.84)	0.03 (0.06)
Contrast 2	0.02 (0.06)	0.37 (0.92)	0.06 (0.07)
Intergroup anxiety		5.73 (0.70) ***	−0.18 (0.05) ***

Notes. In contrast 1, the culturally humble imagined contact condition was coded +2 while the standard imagined contact and the control conditions were coded −1. In contrast 2, the standard imagined contact condition was coded +1, the control condition was coded −1, and the culturally humble imagined contact condition was coded 0. We report unstandardized regression coefficients and, within parentheses, standard errors. * *p* < 0.05. *** *p* < 0.001.

## Data Availability

Data are available at https://doi.org/10.6084/m9.figshare.24492766 (accessed on 3 November 2023).
